# Reconciling patient and provider priorities for improving the care of critically ill patients: A consensus method and qualitative analysis of decision making

**DOI:** 10.1111/hex.12576

**Published:** 2017-05-31

**Authors:** Emily McKenzie, Melissa L. Potestio, Jamie M. Boyd, Daniel J. Niven, Rebecca Brundin‐Mather, Sean M. Bagshaw, Henry T. Stelfox, the Improving Daily Care in the ICU Panel, Jackie de Groot, Caroline Hatcher, Carmen Petersen, Marian Reed, Kathy Sassa, Devina Scholefield, Karrie Whalen, Dan J Zuege, David Zygun

**Affiliations:** ^1^ Alberta Health Services Calgary AB Canada; ^2^ Department of Community Health Sciences University of Calgary Calgary AB Canada; ^3^ Department of Critical Care Medicine University of Calgary Calgary AB Canada; ^4^ W21C Research and Innovation Centre University of Calgary Calgary AB Canada; ^5^ Department of Critical Care Faculty of Medicine and Dentistry University of Alberta Edmonton, Calgary AB Canada

**Keywords:** consensus, critical care, health priorities, intensive care, intensive care unit, patient participation, qualitative research, quality improvement, surveys and questionnaires

## Abstract

**Background:**

Providers have traditionally established priorities for quality improvement; however, patients and their family members have recently become involved in priority setting. Little is known about how to reconcile priorities of different stakeholder groups into a single prioritized list that is actionable for organizations.

**Objective:**

To describe the decision‐making process for establishing consensus used by a diverse panel of stakeholders to reconcile two sets of quality improvement priorities (provider/decision maker priorities n=9; patient/family priorities n=19) into a single prioritized list.

**Design:**

We employed a modified Delphi process with a diverse group of panellists to reconcile priorities for improving care of critically ill patients in the intensive care unit (ICU). Proceedings were audio‐recorded, transcribed and analysed using qualitative content analysis to explore the decision‐making process for establishing consensus.

**Setting and participants:**

Nine panellists including three providers, three decision makers and three family members of previously critically ill patients.

**Results:**

Panellists rated and revised 28 priorities over three rounds of review and reached consensus on the “Top 5” priorities for quality improvement: transition of patient care from ICU to hospital ward; family presence and effective communication; delirium screening and management; early mobilization; and transition of patient care between ICU providers. Four themes were identified as important for establishing consensus: *storytelling* (sharing personal experiences), *amalgamating priorities* (negotiating priority scope), *considering evaluation criteria* and having a *priority champion*.

**Conclusions:**

Our study demonstrates the feasibility of incorporating families of patients into a multistakeholder prioritization exercise. The approach described can be used to guide consensus building and reconcile priorities of diverse stakeholder groups.

## 1 INTRODUCTION

Improving patient safety and quality of care has been at the forefront of health‐care initiatives for decades.^1^, ^2^, ^3^, ^4^, ^5^ The Institute of Medicine has defined quality as “the degree to which healthcare services for individuals and populations increase the likelihood of desired outcomes and are consistent with current professional knowledge.”^6^ High quality care needs to reflect both the clinical and holistic needs of patients.^7^, ^8^ To accomplish meaningful improvements in care, patients, their family members and members of the public need to be engaged in quality improvement initiatives.^9^, ^10^

Identifying priorities for quality improvement has not conventionally involved patients, but this has begun to change with the implementation of patient engagement research strategies in the United Kingdom,^11^ United States^12^ and Canada.^13^, ^14^ For example, patients and families have recently been involved in establishing priorities to inform clinical care in a variety of health conditions including diabetes, stroke, dialysis and eczema.^15^, ^16^, ^17^ However, little is known about how to reconcile the priorities of different stakeholder groups into a single patient‐centred prioritized list that is actionable within the health‐care system.^18^ It is not difficult to imagine that patients and health‐care providers may have different priorities.^10^ For example, patients might consistently rank symptom management or preservation of sleep as a higher priority for improvement than physicians.^19^ Conversely, patients might be unaware of important clinical interventions warranting improvements (eg, venous thromboembolism prophylaxis).^20^

To begin to understand how to best reconcile the priorities of diverse groups of health‐care stakeholders, we undertook a programme of work to establish priorities for improving the care of critically ill patients admitted to intensive care units (ICU). First, health‐care providers (ie, those providers most directly involved in providing patient care in the ICU—physicians, nurses, respiratory therapists and pharmacists) and decision makers (ie, those individuals most directly involved in overseeing patient care in the ICU—unit managers and directors) generated a comprehensive list of candidate daily care practices in the ICU that warrant improvement and subsequently selected nine practices as priorities for quality improvement initiatives.^21^ Second, in an independent process, former patients and family members of patients were engaged to participate in focus groups and interviews to explore their experiences with critical care and identified 19 practices for improvement.^9^ In this manuscript, we report the results of a modified Delphi process that brought together a panel of providers, decision makers and patients’ families to reconcile the two sets of practices (subsequently referred to as priorities) into a single prioritized list. Our primary goal was to describe the decision‐making process, used by a diverse panel of stakeholders that included family members of former ICU patients, to establish consensus for quality improvement priorities.

## 2 METHODS

Using the aforementioned two sets of priorities (1. providers/decision makers^21^ and 2. patients/family^9^), we employed a modified Delphi process with a diverse group of panellists to assess and reconcile priorities for improving care of critically ill patients and used qualitative content analysis to describe the decision‐making process for establishing consensus.

### 2.1 Selection of panellists

Evaluations by other researchers have demonstrated that the composition of consensus panels influences ratings, but the optimal composition and number of panellists have yet to be established.^22^, ^23^, ^24^, ^25^ We assembled a panel of nine individuals representing providers (n=3), decision makers (n=3) and patients’ families (n=3) from ICUs within a single geographically defined health‐care system (Alberta Health Services, Alberta, Canada) responsible for providing integrated health‐care services to a population of 4.2 million residents.^26^ We elected to comprise the panel equally of these three stakeholder groups given our perspective that each group is equally important for health‐care quality (delivering care, overseeing care delivery, receiving care).^7^, ^10^ Panellists were purposively nominated by the leadership of the health‐care system's Critical Care Strategic Clinical Network (CCSCN), a province‐wide network of critical care medicine stakeholders to ensure representation of different experiences, expertise and geographical locations (Table S1).^9^, ^27^

### 2.2 Rating instrument

We developed an electronic survey instrument that was a summation of the priorities selected for quality improvement (Table S2) by provider/decision makers (n=9)^21^ and patients and their family members (n=19).^9^ The instrument presented a description of each priority across six criteria: strength of the evidence in the literature; impact on costs; an actionable practice change; easily measurable results; potential to benefit/harm patients; and impact on patient/family experience. We then asked panellists to select their “Top 10” priorities for improvement.

### 2.3 Priority rating process

The ratings were conducted using a three‐phase modified Delphi process in which the panellists conducted two rounds of remote review of the candidate priorities using secure electronic survey instruments (Rounds 1 and 2 [see Table S3, ICU Priorities Rating Tool]), followed by an in‐person meeting (Round 3) during which there were two rounds of voting (Rounds 3.1 and 3.2) to select the final priorities for improvement. During Round 1, panellists were asked to select their “Top 10” among the 28 priorities identified for quality improvement. During Round 2, panellists were provided with the number of panellists that selected each priority as in the “Top 10” from the previous round and asked to rate the priorities again.

Round 3 was a half‐day in‐person meeting, facilitated by an experienced facilitator, held in Calgary, Alberta, on 14 November 2014. The goal of the meeting was to reach consensus on the “Top 5” priorities for quality improvement that represented the perspectives of the three stakeholder groups. The day opened with an explanation of meeting objectives, an overview of the priority‐setting process and a presentation of the findings from the first two rounds of remote voting by the panellists. Over the course of the day, panellists completed two further rounds of voting. Flipcharts were dedicated to single priorities, with a definition and descriptors provided. Panellists were given five stickers and asked to use them to indicate their “Top 5” priorities. A round‐table discussion of each priority and the rationale for keeping or removing it occurred between the two rounds of voting.

### 2.4 Analysis

#### 2.4.1 Ratings of priorities for improvement

Panellists’ ratings of the reconciled list of priorities were summarized using counts. Priorities were selected for further consideration after each round of deliberation based on frequency counts. Priorities selected by all three panellists from a given stakeholder group (provider, decision maker, family), regardless of the other panellists’ ratings, were advanced for further deliberation. This was designed to ensure that when a stakeholder group was unanimous in selecting a given priority, the other stakeholders could not eliminate it. Based on panellists’ feedback in Round 1, the priorities were amalgamated and/or revised prior to Round 2.

#### 2.4.2 Thematic analysis of stakeholder discussion

The in‐person meeting was audio‐recorded and transcribed verbatim for the purpose of content analysis. One researcher (RBM) recorded qualitative observations and memos during the event. Two researchers (EM, MLP) independently conducted a conventional qualitative content analysis of the data.^28^, ^29^ Applying the methods as outlined by Miles and Huberman,^30^ the inductive analysis process involved allowing the codes, categories and themes to be directly derived from the text transcript. Comparisons between codes were made to identify similar codes and to allow them to be sorted into categories.^31^ Any disagreements were resolved by consensus. The descriptive themes were designed to explain the text within the categories and highlight key factors informing the panellists’ decision‐making process. Disfluencies were removed from exemplar quotes to facilitate readability.

### 2.5 Ethics

We obtained written consent from all participants to audio‐record the proceedings. The Conjoint Health Research Ethics Board, University of Calgary (REB13‐1157), approved this study.

## 3 RESULTS

### 3.1 Modified Delphi process and resulting priority ratings

**Figure 1:**
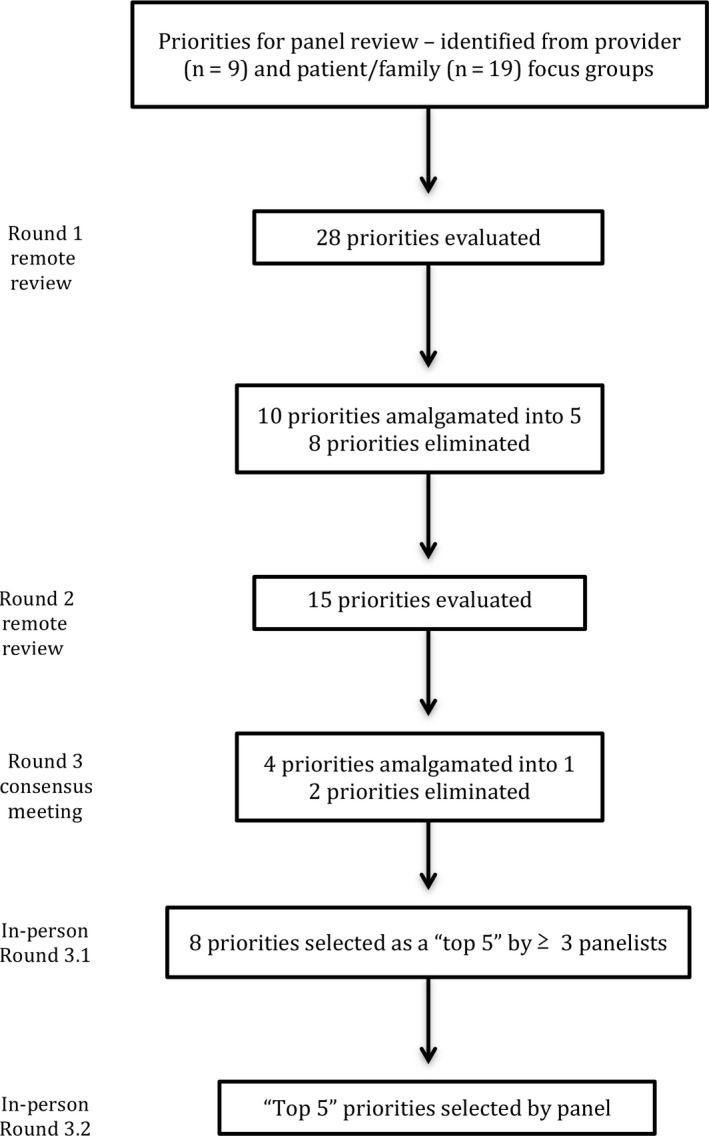
Flow of priorities through prioritization rounds

We invited nine individuals to participate in the consensus process, and all nine individuals agreed to participate (participation rate: 100%). The flow of the priorities across the three rounds of panel review during the modified Delphi process is depicted in Figure 1. Panellists were presented with 28 priorities (1. 9 provider/decision‐maker priorities, 2. 19 patient/family priorities) in Round 1 and 15 priorities in Round 2. Based on panellists’ ratings and feedback over three rounds of review, 10 priorities were eliminated, 13 priorities were amalgamated into 5 priorities, and the panellists came to consensus on the “Top 5” priorities (Table 1) for quality improvement. Table S4 (Panellists’ Ratings Across Rounds) summarizes the panellists’ ratings and the amalgamation and removal of priorities across the rounds of the modified Delphi process.

**Table 1:** Top five priorities for quality improvement

Top rated priorities	No. of panellists selecting the priority (N=9)
Transition of patient care from ICU to hospital ward	9
Family presence and effective communication	8
Delirium screening & diagnosis	8
Early mobilization	7
Transition of patient care between providers within ICU	6

### 3.2 Content analysis of the decision making and consensus process

Four main themes related to the decision‐making process to establish consensus for priorities for quality improvement were identified from the audio data and notes collected during the in‐person meeting: (i) storytelling, or sharing personal experiences; (ii) amalgamating or negotiating the scope of focus; (iii) consideration of evaluation criteria (ie, strength of the evidence, impact on costs, actionable, measurable, potential benefit/harm, patient/family experience); and (iv) having a priority champion (Table 2).

**Table 2:** Main themes from observed approaches to decision making for establishing consensus

Theme	Description
Storytelling (sharing personal experiences)	Stories or personal experiences shared by panellists to provide a deeper understanding of one's position
Amalgamating priorities (negotiating the scope)	Adjust or combine like‐priorities to provide clarity and definition of scope
Consideration of evaluation criteria (ie, strength of the evidence, impact on costs, actionable, measurable, potential benefit/harm, patient/family experience)	A standard framework of pre‐specified criteria to consider when evaluating the priority to facilitate decision making around the rating
Having a priority champion	Declare whether or not he/she selected a priority and describe the rationale and justification for their decision.

#### 3.2.1 Storytelling or sharing personal experiences

“*Storytelling or sharing personal experiences*” was an important contributor to how decisions were made during the consensus process. During round‐table discussions, several panellists, including family members and providers, shared personal experiences. A panellist described a lengthy wait as his/her daughter was undergoing an elective procedure, after which the panellist and his/her family had poor communication with the surgeon, and how this resulted in anxiety. The panellist juxtaposed the experience, which involved minor elective surgery to that of families of critically ill ICU patients and stressed the importance of *family presence and effective communication*. The sharing of personal experiences contextualized the concept of gaps between what best evidence suggests patients should receive and what they actually receive as care (evidence‐care gap)^2^ facilitating consensus among the panellists (see Table S4, Panellists’ Ratings Across Rounds).

“And so eventually at about an hour and 45 minutes, the surgeon comes out and I knew exactly what happened ‘cause he passed [us] to talk to another family first. So he did another case without coming out [to speak with us]… my wife was decompensating, I was a little bit concerned. I couldn't imagine if my child or my loved one was actually in a life or death situation, not having information immediately’’.

#### 3.2.2 Amalgamating or negotiating the scope of focus

*“Amalgamating or negotiating the scope of focus”* was a critical strategy used by the panellists in moving towards consensus in their decision‐making process. For example, panellists began the in‐person meeting by suggesting that several priorities be amalgamated prior to the first in‐person voting round (*family is patient's voice; inviting family to be part of care team; discussions of prognosis; and timely updates for major changes*) to form a single priority entitled, “*family presence and effective communication*” (see Table S4, Panellists’ Ratings Across Rounds). Panellists felt that the four separate priorities created artificial divisions across a shared construct and that amalgamating them into a single priority would support meaningful action for quality improvement. These four priorities were originally identified through patient and family focus groups.^9^ Merging the four priorities into a single priority also served the strategic purpose of ensuring that the patient/family voice (ie priority) was selected as a “Top 5” priority. The round‐table discussion allowed panel members to reach consensus on the scope of the priorities. For example, panellists did not originally agree that delirium screening was an important priority. However, after discussing the interrelationship between sleep, early mobilization, delirium and patient outcomes, the priority was modified to include both delirium screening and management and selected as a “Top 5” priority. Amalgamating priorities and negotiating their scope were perceived by panellists to be an important strategy to avoid unintentional vote splitting on important and inter‐related priorities.

When I was forced to vote for one, I see patient as family voice, I see discussion with family in inviting family to be part of the team, and I see keeping families informed. It's hard for me to vote for any one of those to prioritize, and I think it's probably family resonated but you actually split up votes. [That should all be] one category, and it's not just [about] keeping them informed but [also] having them inform us.

#### 3.2.3 Consideration of evaluation criteria (ie, strength of the evidence, impact on costs, actionable, measurable, potential benefit/harm, patient/family experience)

“*Consideration of evaluation criteria*” was frequently important in guiding panellists towards consensus. Panellists continually revisited and debated the strength of the evidence associated with a given priority, considered how readily action could be taken to improve the practice and finally how change could be measured. For example, panel members agreed that while *discussions of prognosis* are important, it was perceived that both meaningful action to improve the practice and accurate measurement were unlikely, and thus, panellists came to the consensus to not include it as a high priority.

Well I've been one of the people that didn't rank that actually and it's certainly not reflective of its importance. It's being a little less certain of where the gap is and how measurable and actionable it is. You know the thing, if there [are] gaps, it's along the lines of people don't want to talk about it or how effectively they talk about it, and that's just a lot softer and I think it's harder to address and it certainly isn't reflective of importance or that there is a gap, I just, I wonder how actionable and measurable it is.

#### 3.2.4 Having a priority champion

“*Having a priority champion”* was important in driving the panellists to consensus. The panellists were all comfortable declaring their “position” on each of the priorities. During the discussion, panellists would declare whether or not they selected a particular practice as a priority for quality improvement and would describe the rationale for their decision. They demonstrated a willingness to share with others, and the result was a respectful and insightful conversation. This open sharing of viewpoints fostered efficient and effective consensus building.

I would have voted for this. I guess you know part of the trouble for me is distinguishing whether daily patient goals are established, which I think they are, and how effectively, or where they're being documented. I actually don't have too much doubt that goals are established; they may not be documented in a consistent place… So I didn't vote for it because I think, I think the more important part of that is that they are established and a little less the documentation and where.

## 4 DISCUSSION

We successfully conducted a consensus process involving an expert panel of providers, decision makers and family members of past ICU patients to reconcile priorities for quality improvement independently developed by two different stakeholder groups into a single joint prioritized list. Qualitative content analysis of panel discussions and deliberations identified four themes central to the process of decision making for establishing consensus: storytelling (sharing personal experiences), amalgamating priorities (negotiating the scope), consideration of evaluation criteria, and having a priority champion. The priorities can be used to guide quality improvement initiatives and the themes to guide consensus methods.

There has been significant investment in priority setting across diverse fields in health‐care employing different methods including expert panels, focus groups, voting surveys, interviews and consensus meetings.^15^, ^16^, ^32^, ^33^, ^34^ The James Lind Alliance advocates for partnerships between clinicians and patients in priority setting to ensure that the needs of both groups are represented and to help establish a framework for action.^8^ For example, the James Lind Alliance has facilitated over 70 partnerships to develop prioritized lists for health research. However, individual stakeholder groups may nevertheless have distinct priorities and reconciliation is necessary to allow for focused action.^18^ To the best of our knowledge, the process of reconciling the priorities of different stakeholders has received little attention and needs to be evaluated.^18^ Evaluations by other researchers have demonstrated that the composition of consensus panels influences the ratings. There are differences in judgement based on physician specialty,^35^ between mixed‐ and single‐specialty physician panels^23^, ^24^ and between mixed physician and non‐physician panels.^25^ Our study demonstrates that it is feasible for a panel of diverse stakeholders representing providers, decision makers and patient families to review, discuss and reach a consensus regarding priorities for quality improvement. The modified Delphi approach employed allowed us to combine a highly structured anonymous process for rating the priorities and an interactive meeting to explore areas of disagreement.^36^ The qualitative analysis of the consensus meeting identified four themes that summarize key strategies employed by stakeholders to establish consensus.

*Storytelling* is a universal form of communication: stories are creative representations of a person's experiences.^37^ Whether communicated verbally or in writing, storytelling is the sharing of a personal narrative^37^ and is commonly used in sociological and anthropological research.^38^, ^39^ Stories can be used to pique interest and enable the storyteller to connect with their audience on an emotional level. They help convey key information in a memorable way and thus help the storyteller to persuade their audience to take action. In our study, storytelling was observed to foster decision making by capturing the panel's attention and engaging them in discussion, resulting in panellists having a deeper understanding of the storyteller's position.

*Amalgamating or negotiating the scope* of a priority can be important in establishing consensus.^40^ It allows for similar concepts to be brought together. However, panellists’ must be cognizant to avoid developing a concept that is too broad or vague (ie, too many dimensions) and subsequently hard to act on.^7^ In a qualitative analysis of a consensus process to develop quality indicators of injury care, Bobrovitz et al. reported that clarifying the scope and goals of a practice was a prominent factor in establishing consensus.^40^ This observation was reflected in our study with face‐to‐face dialogue allowing panellists to pose questions and receive clarification around priority scope and definition, before making their final selections.

*Consideration of criteria* is essential in focusing a discussion towards consensus.^40^ Criteria provide panellists with a standard framework from which they can consider pre‐specified elements of the priority and rate it accordingly.^22^ Previous prioritization and consensus studies have successfully utilized a specified set of criteria to facilitate the decision‐making process.^41^, ^42^, ^43^ Our study further demonstrates how panellists recurrently return to the criteria to guide decision making. This highlights the importance of careful consideration when selecting evaluation criteria for a consensus process.

*Champions* influence the selection of priorities. For example, Hutten et al. conducted a priority‐setting study on psychological treatments and services for people experiencing longer‐term depression using multiple stakeholder views.^18^ Ideas for service improvement were presented at a consensus workshop and through a series of “idea champions.” Idea champions recognized the value of the idea and attempted to convey this value to others. The researchers^18^ identified individuals to champion specific ideas a priori, whereas idea champions (akin to *priority champions*) naturally evolved during the round‐table discussions in our consensus workshop, helping to galvanize panellists to consensus. The use of idea champions needs to be carefully managed and monitored to mitigate the introduction of biases during group decision making, such as “groupthink,” which occur in situations where individuals avoid raising controversial issues.^44^ Anonymous rounds of in‐person voting and moderation of the discussion by a trained and experienced facilitator (both used in our study) are two strategies to manage this risk.^18^

The results of our study should be interpreted in the context of its strengths and limitations. Three primary limitations should be considered. First, our panellists were purposefully selected from participants in previous phases of the research programme. Although participants represented different constituents and geographical regions, their views may not be representative of the broader ICU provider, decision maker, family and patient population. However, the original list of 28 priorities shared with panellists at the start of the consensus process was developed through a population‐based initiative that incorporated the perspectives of 1,135 providers and decision makers and 32 patients and family members. Second, our panel membership did not include an ICU survivor. The severity and complexity of illness warranting care in the ICU often limits patient participation in care.^45^, ^46^, ^47^ As such, it is often family members who act as the patient's voice to communicate their wishes and hence are often to best ones to recall the patient's care journey.^48^, ^49^ Finally, the research was conducted using a single consensus process involving nine panel members in a single health‐care system and was restricted to ICU services. It is possible that the reconciliation process might be different if replicated with additional stakeholder panels in other health‐care systems or health‐care domains.

## 5 CONCLUSION

Historically, providers and decision makers have established priorities for quality improvement. Our study demonstrates the feasibility of engaging the families of patients in this process and achieving consensus among a diverse group of stakeholders. Storytelling (sharing personal experiences), amalgamating priorities (negotiating the scope), consideration of evaluation criteria and having a priority champion appear to be important for establishing consensus. The approach employed in our study can be used to establish consensus among diverse stakeholder groups.

## CONFLICTS OF INTEREST

None to declare.

## AUTHORS' CONTRIBUTIONS

All authors (EM, MLP, JMB, DJN, RBM, SMB, JDE, CH, CP, MR, KS, DS, KW, DJZ, DZ, HTS) contributed to the study's conception, design and interpretation. EM and MLP conducted the analyses. EM and JMB drafted the manuscript, and all authors (EM, MLP, JMB, DJN, RBM, SMB, JDE, CH, CP, MR, KS, DS, KW, DJZ, DZ, HTS) assisted in the successive revisions of the final manuscript. SMB and HTS secured funding for the work. The final version has been seen and reviewed by all authors (EM, MLP, JMB, DJN, RBM, SMB, JDE, CH, CP, MR, KS, DS, KW, DJZ, DZ, HTS), and they assume responsibility for the integrity of the data and the accuracy of the analysis.
