# Supplementary material for: Reconciling patient and provider priorities for improving the care of critically ill patients: A consensus method and qualitative analysis of decision making
Source: Health Expect. 2017 May 31;20(6):1367–74. doi: 10.1111/hex.12576 (PMC5689241; doi:10.1111/hex.12576)
Supplement: Supplementary file 1 [file HEX-20-1367-s001.docx]

Table S1. Reconciliation Panel Members

| **Panellist^*^ Role** | **ICU Expertise/Experience** |
| --- | --- |
| Decision-maker | Clinical Manager |
| Decision-maker | Executive Director |
| Decision-maker | Medical Director |
| Provider | Physician |
| Provider | Respiratory Therapist |
| Provider | Nurse |
| Family member | Common Law spouse had pneumonia with H1N1 |
| Family member | Son was born with illness that necessitated intermittent ICU stays over a two decade period |
| Family member | Husband was in ICU twice over multi-month period |

^*^Panellists recruited from six different cities.
