# Supplementary material for: Reconciling patient and provider priorities for improving the care of critically ill patients: A consensus method and qualitative analysis of decision making
Source: Health Expect. 2017 May 31;20(6):1367–74. doi: 10.1111/hex.12576 (PMC5689241; doi:10.1111/hex.12576)
Supplement: Supplementary file 2 [file HEX-20-1367-s002.docx]

Table S2: Provider and Patient/Family Member Priorities

| **Frontline Provider Priorities** | **Patient and Family Member Priorities** |
| --- | --- |
| Daily patient care goals | Patient and family transition into ICU |
| Daily sedation interruption | Family shock and disorientation |
| Delirium screening & diagnosis | Presence and support of a provider |
| Early mobilization | Patient’s (in)ability to communicate |
| End-of-life care | Family is the patient’s voice |
| Strategies to preserve patient sleep | Daily updates |
| Temperature control in patients after resuscitation from cardiac arrest | Timely updates for major changes |
| Transition of patient care between providers within ICU | Keeping patient information private |
| Transition of patient care from ICU to hospital ward | Discussions of prognosis |
|  | Balance of hope and reality |
|  | Goals of care |
|  | Providing the best medical care |
|  | Continuity of providers |
|  | Access to support |
|  | Inviting family to be part of the care team |
|  | Allowing family to be with the patient |
|  | ICU facilities for families |
|  | Transition from ICU to a hospital ward |
|  | Long-term effects of critical illness |
