# Supplementary material for: Reconciling patient and provider priorities for improving the care of critically ill patients: A consensus method and qualitative analysis of decision making
Source: Health Expect. 2017 May 31;20(6):1367–74. doi: 10.1111/hex.12576 (PMC5689241; doi:10.1111/hex.12576)
Supplement: Supplementary file 3 [file HEX-20-1367-s003.pdf]

Supplementary File 3

Priority Rating Tool (with sample Priority)

PROVIDER Generated Priorities

3. Delirium screening & diagnosis (using a validated tool to routinely screen patients for the presence of delirium)

Synthesis of best available information
(presented during Provider rating process):

**Strength of Evidence**	High
**Cost**	Lower
**Measurable**	High
**Actionable**	Likely
**Potential to Benefit/Harm Patient**	Unknown
**Patient/Family Experience**	Unknown

Reconciliation Process: Improving Daily Care in the ICU

PLEASE PICK 10 OF THE FOLLOWING AS YOUR TOP CHOICES

Please select 10 of the following that you think are most important. It is acceptable to choose fewer than 10.

- [ ] Daily patient care goals
- [ ] Daily sedation interruption
- [ ] Delirium screening & diagnosis
- [ ] Early mobilization
- [ ] End-of-life care
- [ ] Strategies to preserve patient sleep
- [ ] Temperature control in patients after resuscitation from cardiac arrest
- [ ] Transition of patient care between providers within the ICU
- [ ] Transition of patient care from ICU to hospital ward
- [ ] Patient and family transition into ICU
- [ ] Family shock and disorientation
- [ ] Presence and support of a provider
- [ ] Patient's (in)ability to communicate
- [ ] Family is patient's voice
- [ ] Daily updates
- [ ] Timely updates for major changes
- [ ] Keeping patient information private
- [ ] Discussions of prognosis
- [ ] Balance of hope and reality
- [ ] Goals of care
- [ ] Providing the best medical care
- [ ] Continuity of providers
- [ ] Access to support
- [ ] Inviting family to be part of the care team
- [ ] Allowing family to be with the patient
- [ ] ICU facilities for families
- [ ] Transition from ICU to a hospital ward
- [ ] Long-term effects of critical illness

Reconciliation Process Round 2: Improving Daily Care in the ICU

Potential Priority for Quality Improvement

3. Delirium screening & diagnosis (using a validated tool to routinely screen patients for the presence of delirium)

Synthesis of best available information (presented during Provider rating process):

**Strength of Evidence**	High
**Cost**	Lower
**Measurable**	High
**Actionable**	Likely
**Potential to Benefit/Harm Patient**	Unknown
**Patient/Family Experience**	Unknown

Number of panelists who selected this priority: 6

Reconciliation Process Round 2: Improving Daily Care in the ICU

Please pick 10 of the following as your top choices for improving care:

- [ ] Transition of patient care from ICU to hospital ward **(newly formed: combined with patient and family transition from ICU to hospital ward)**
- [ ] Early mobilization
- [ ] Delirium screening
- [ ] Prognosis discussions and establishing goals of care **(newly formed: combined discussions of prognosis AND goals of care)**
- [ ] Daily patient care goals
- [ ] Immediate and ongoing communication with family about patient status and ICU culture, as well as family support
- [ ] End-of-life care
- [ ] Transition of patient care between providers within ICU **(newly formed: combined transition of patient care between providers within ICU AND continuity of staff)**
- [ ] Keeping families informed **(newly formed: combined daily updates AND timely updates for major changes)**
- [ ] Daily sedation interruption
- [ ] Providing the best medical care
- [ ] Strategies to preserve patient sleep
- [ ] Patient and family transition into ICU **(newly formed: combined patient and family transition into ICU AND family shock and disorientation)**
- [ ] Family is the patient's voice
- [ ] Inviting family to be part of the care team

Do you have any comments or suggestions?
