# Supplementary material for: Reconciling patient and provider priorities for improving the care of critically ill patients: A consensus method and qualitative analysis of decision making
Source: Health Expect. 2017 May 31;20(6):1367–74. doi: 10.1111/hex.12576 (PMC5689241; doi:10.1111/hex.12576)
Supplement: Supplementary file 4 [file HEX-20-1367-s004.docx]

Table S4. Panelists’ Ratings Across Rounds^1,2^

| Priority^2^ | **e-Survey Voting**  **(Number of Panelists who included priority in ‘Top 10’)** | | **In-Person Meeting**  **(Number of Panelists who included priority in ‘Top 5’)** | |
| --- | --- | --- | --- | --- |
|  | **Round 1** | **Round 2** | **Round 3.1** | **Round 3.2** |
| **Transition of Patient Care from ICU to Hospital Ward**^3^ |  | 8 | **4** | **9** |
| Patient and Family Transition from ICU to Hospital Ward | 8 |  |  |  |
| Transition of Patient Care from ICU to Hospital Ward | 6 |  |  |  |
| **Family Presence and Effective Communication**^4^ |  |  | **8** | **8** |
| Family is the Patient’s Voice | 3 | 2 |  |  |
| Inviting Family to be Part of Care Team | 3 | 5 |  |  |
| Ongoing Communication with Family re: Patient status & ICU Culture | 5 | 5 |  |  |
| **Keeping Families Informed**^3^ |  | 8 |  |  |
| Day-to-day Care Updates | 3 |  |  |  |
| Timely Updates on Major Changes When Absent from ICU | 3 |  |  |  |
| **Delirium Screening** | 6 | 6 | **4** | **8** |
| **Early Mobilization** | 6 | 7 | **5** | **7** |
| **Transition of Patient Care Between Providers Within ICU**^3^ |  | 7 | **3** | **6** |
| Continuity of Staff | 2 |  |  |  |
| Transition of Patient Care Between Providers Within ICU | 4 |  |  |  |
| **Prognosis Discussions & Establishing Goals of Care**^3^ |  | 9 | 6 | 2 |
| Prognosis Discussions | 4 |  |  |  |
| Goals of Care | 3 |  |  |  |
| **Daily Patient Care Goals** | 5 | 7 | 5 | 1 |
| **Patient and Family Transition into ICU**^3^ |  | 6 | 3 | 1 |
| Patient and Family (Dis)Orientation | 1 |  |  |  |
| Patient and Family Transition into ICU | 3 |  |  |  |
| **End-of-Life Care** | 5 | **6** | 1 | 1 |
| **Strategies to Preserve Patient Sleep** | 4 | **6** | 1 | 0 |
| **Providing Best Medical Care** | 4 | **5** |  |  |
| **Daily Sedation Interruption** | 4 | **3** |  |  |
| **Balance of Hope and Reality** | **2** |  |  |  |
| **ICU Facilities for Families** | **2** |  |  |  |
| **Keeping Patient Information Private** | **2** |  |  |  |
| **Allowing Family to be with Patient as Needed** | **1** |  |  |  |
| **Post-ICU Concerns When Patient is Home** | **1** |  |  |  |
| **Patient’s (In)ability to Communicate** | **0** |  |  |  |
| **Provide Ongoing Access to ICU Support Staff** | **0** |  |  |  |
| **Temperature in Patients After Resuscitation from Cardiac Arrest** | **0** |  |  |  |

^1^ Grey shaded cells indicate the priority was not rated that round because it had been previously eliminated according to ratings or was added by the panel during that round.

^2^ Priorities listed according to frequency counts of panelist votes with amalgamated priorities clustered together

^3^ Based on Round 1 feedback, priorities were amalgamated into a new priority for further evaluation.

^4^ Based on Round 2 feedback & Round 3 discussion, one previously amalgamated priority and three additional priorities were amalgamated into a single new priority prior to In Person voting Round 3.1.
